# Lyme Disease Presenting as Rupture Baker’s Cyst: Case Report and Literature Review

**DOI:** 10.1177/11795476261457180

**Published:** 2026-05-31

**Authors:** Abdullah Ghali, Neal Mody-Bailey, Mohamad Jabin, Marwa Abdou, Momin Hussain, Amr Abdelgawad

**Affiliations:** 1 Department of Orthopaedics, 3989 Baylor College of Medicine, Houston, TX, USA; 2 Texas A&M School of Medicine, Dallas, TX, USA; 3 Department of Pediatrics, 24513 Kings County Hospital, Brooklyn, NY, USA; 4 Long School of Medicine, UT Health San Antonio, San Antonio, TX, USA; 5 Department of Orthopaedics, 2042 Maimonides Medical Center, Brooklyn, NY, USA

**Keywords:** lyme disease, tick-borne diseases, ticks, orthopaedics/rehabilitation/occupational therapy, infectious diseases

## Abstract

Baker’s cysts, also known as popliteal cysts, are fluid-filled sacs that are found in the posterior region of the knee, behind the medial head of the gastrocnemius and the semimembranosus. The prevalence has been found to increase with age, with some ultrasonographic studies showing a prevalence as high as 25% of adults with knee pain. We discuss an interesting case of a 14-year- old male with a ruptured cyst found to be secondary to Lyme disease. The patient was treated with clindamycin and his symptoms had resolved. We discuss cases of ruptured baker’s cysts in the literature found to be secondary to Lyme disease with their respective treatments and outcomes.

## Introduction

Baker’s cysts, also known as popliteal cysts, often resulting from distension of the bursa between the medial gastrocnemius muscle and the semimembranosus muscle.^1^ Its pathophysiology generally involves communication between the knee joint and the bursa, facilitated by a one-way valve mechanism that allows synovial fluid to accumulate in the bursa during intra-articular effusion, often secondary to a knee condition such as osteoarthritis, meniscal injury, or inflammatory arthritis.^1^ The prevalence has been found to increase with age, with some ultrasonographic studies showing a prevalence as high as 25% of adults with knee pain^2^.

In children, Baker’s cysts are most often primary conditions that herniate from the posterior knee joint capsule.^3^ These cysts can have a broad differential diagnosis from infectious, traumatic or inflammatory diagnoses.^4^ While popliteal cysts are common in the pediatric population, their presentation as a manifestation of Lyme disease is exceedingly rare.^4^ In addition, occasionally this baker cyst due to Lyme disease can rupture causing severe pain and swelling in the posterior knee and calf region. Due to the rarity of this condition, children with ruptured Baker’s cysts due to Lyme disease may be harder to diagnose.

The purpose of this study is to present our case of a ruptured Baker’s cyst in the setting of Lyme disease presenting with ruptured Baker’ cyst and to present a systemic review of the literature of this rare condition.

## Methods

### Case Description

**Figure 1.**
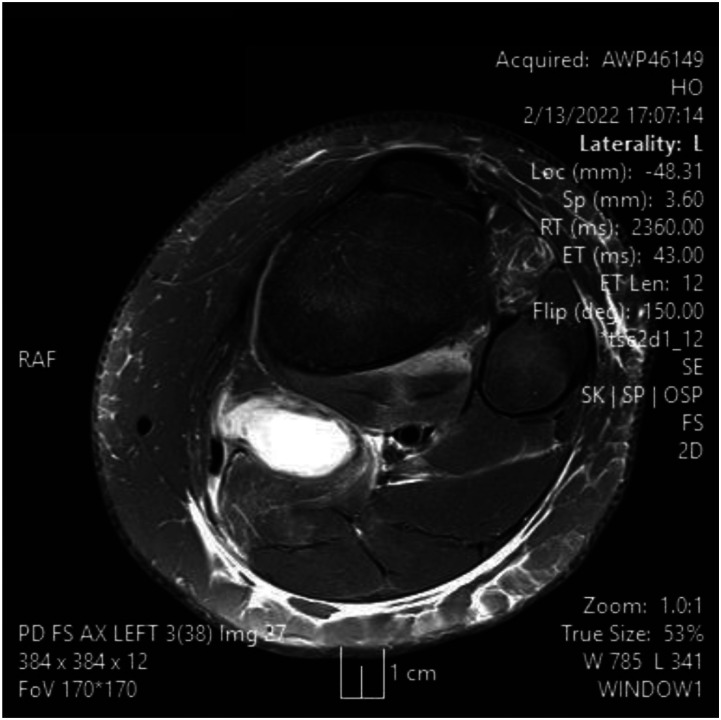
MRI slices on T2 signal showing increased signal of Left knee popliteal bursa. Fig 1 shows an axial slice

**Figure 2.**
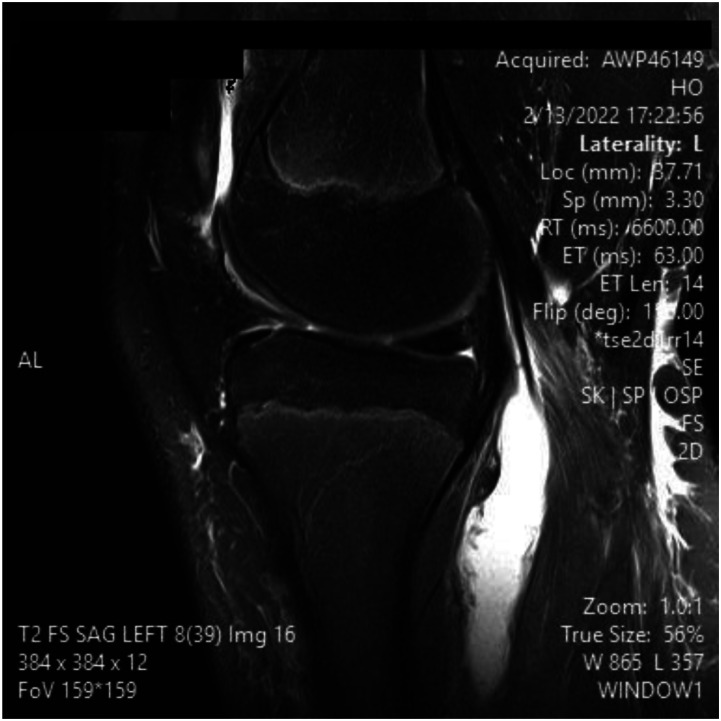
MRI slices on T2 signal showing increased signal of Left knee popliteal bursa. Figure 2 show a sagital slice

**Figure 3.**
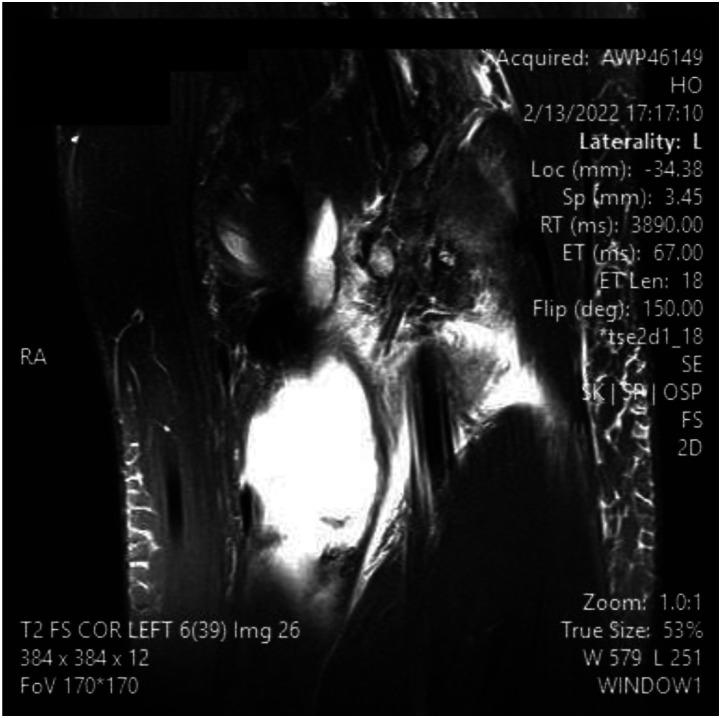
MRI slices on T2 signal showing increased signal of Left knee popliteal bursa. Figure 3 shows a coronal slice

**Figure 4.**
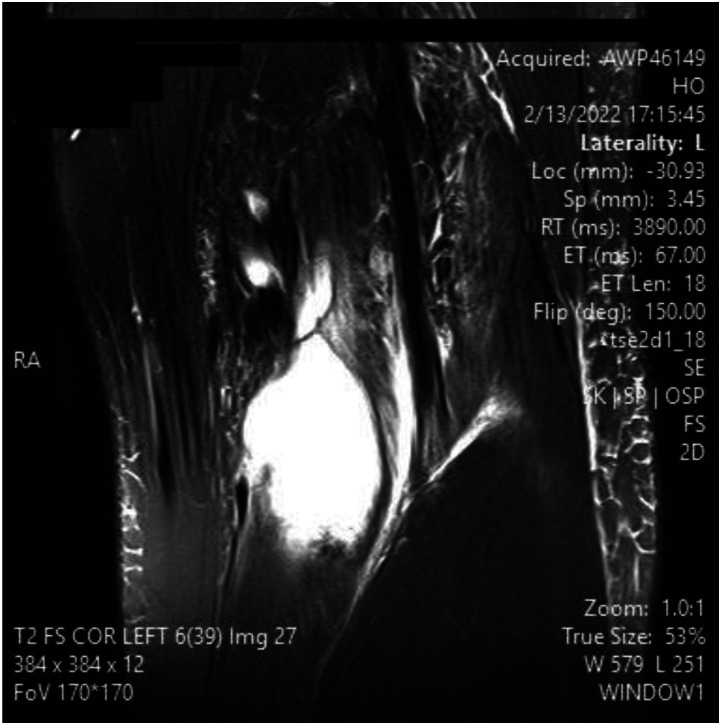
MRI slices on T2 signal showing increased signal of Left knee popliteal bursa. Figure 4 shows a coronal slice

**Figure 5.**
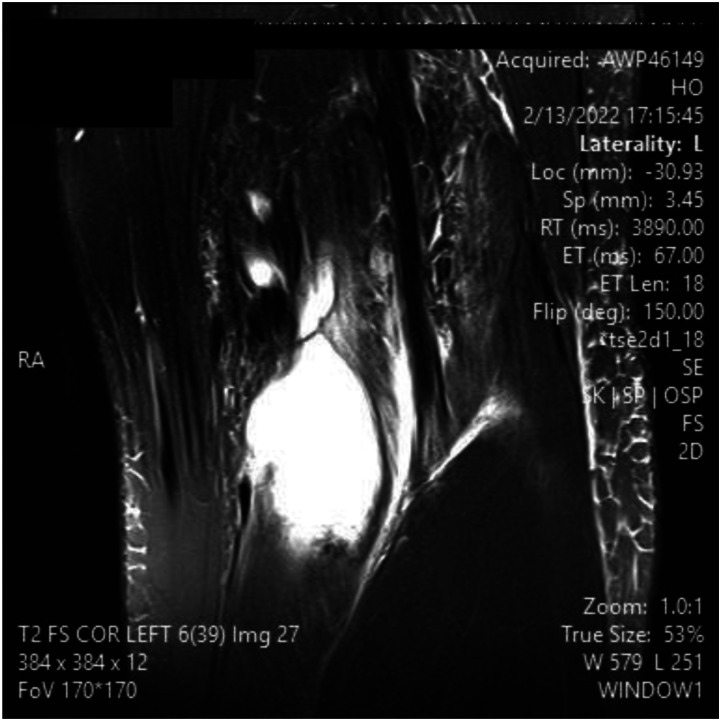
MRI slices on T2 signal showing increased signal of Left knee popliteal bursa. Figure 5 shows a coronal slice

A 14 y/o male presented after one week of left knee pain and swelling with new added pain and swelling in the lower calf. Upon examination, the patient was found to have swelling in the posterior border of the knee and calf. The patient endorsed a history of travel to a Lyme disease endemic area. A decision was made to draw inflammatory markers as well as serology with ELISA and West Blot. Serology was positive for Lyme disease with lyme antibody screen with reflex of confirmation and elevated Lyme IgG along with elevated ESR/CRP, seen in appendix 1. MRI was ordered as shown in Figures 1-5. It showed high intensity on T2 signal for bursal fluid in the popliteal area, capturing a Baker’s cyst with fluid dissecting through the medial head of Gastrocnemius muscle representing indicating rupture Baker Cyst. The patient was started on Clindamycin and then transitioned to doxycycline for 4 weeks after serology confirmed Lyme disease, leading to complete resolution of his symptoms including swelling and pain. Upon follow up, the patient did not report recurrence of his symptoms.

### Study selection

A search was conducted using the Medline database on PubMed, Google Scholar, and Science Direct for studies describing ruptured Baker’s cysts in Lyme disease. The articles were screened for patients with ruptured Baker’s cysts who had confirmatory studies outlining the cause to be Lyme disease. If there was a question about the study qualification, a second and more senior reviewer assessed the article. After the exclusion process, the number of eligible studies was 5 with 8 reported retrospective cases.^4-8^ Data were independently extracted by two reviewers and crosschecked. The data was stored on an excel sheet.

Primary MEDLINE search for keywords Lyme; Baker’s cyst; popliteal cyst; ruptured; borrelia burgdorferi; systematic review.

## Results

A total of eight patients are documented in the literature with Lyme disease ruptured Baker’s cysts. All patients in our case report and in the literature review were male with an average age of 30.8 years old and with a range between 7-59 years old. The average symptom duration was 5.25 days. Four patients underwent surgical debridement with antibiotics and four patients were only treated with antibiotics. All patient clinical characteristics are displayed in Table 1. All patients had negative fluid cultures. Seven of the Eight patients were either in Lyme disease endemic areas or had been a boy scout camping in a Lyme disease endemic area.

**Table 1.** Summary of 8 Patient With Ruptured Baker’s Cysts Secondary to Lyme Disease. Table Included Treatment, Outcome and Publication Hometown

Patient	Age/Sex	Symptom duration	Fluid culture	Lyme testing	Treatment	Outcome	Publication hometown
1	7 yo M	4 days	None	PCR +	Surgical Arthrotomy with Vancomycin and Clindamycin; 5 day course	No Response	Minneapolis, MN (boyscout in Lyme endemic area)
​				MRI	Amoxicillin; 28 day course	Resolution	​
2	15 yo M	7 days	None	WB +	Arthroscopic Debridement with Cefazolin; 5 day course	No Response	Minneapolis, MN (boyscout in Lyme endemic area)
​				MRI	Doxycycline; 8 week course	Resolution	​
3	14 yo M	2 days	None	PCR +	Surgical Arthrotomy with Doxycycline; 1 month course	Resolution	Minneapolis, MN (boyscout in Lyme endemic area)
​				US then MRI	​	​	​
4	54 yo M	6 days	None	ELISA +/WB +	Ceftriaxone; 4 week course	Resolution	New York City, NY
​				MRI	​	​	​
5	59 yo M	7 days	None	ELISA +/WB + /PCR +	Ceftriaxone; 4 week course	Resolution	New York City, NY
​				MRI	​	​	​
6	52 yo M	2 days	None	ELISA +/WB + /PCR +	Cefazolin, Levofloxacin, Vancomycin, Clindamycin	No Response	Camden, New Jersey
​				MRI	Ceftriaxone; 3 week course	Resolution	​
7	8 yo M	7 days	None	ELISA +/WB +	Cefazolin; prior to operation	Resolution	Philadelphia, PA
​				MRI showing Pseudothromboph lebitis from ruptured Baker’s cyst	Amoxicillin; 4 week course	Resolution	​
8	38 yo M	7 days	None	ELISA +	Ceftriaxone; 3 week course	Resolution	Paris, France
​				US showing rupture cyst with sural phlebitis	​		

## Discussion

Lyme arthritis, especially in the pediatric population, can present as acute nontraumatic joint swelling, with the knee being the most common site of infection.^9,10^ This clinical presentation in children can often be mistaken as JIA (Juvenile Idiopathic Arthritis, previously known as Juvenile Rheumatoid Arthritis). As the disease process develops, a popliteal cyst may form and lead to various complications. These include rupture, dissection, compartment syndrome, compression syndrome, or pseudothrombophlebitis.^11,12^ Rupture of a baker’s cyst into the gastrocnemius may simulate deep vein thrombosis symptoms and/or myositis symptoms such as acute pain, swelling, and redness. Pain and swelling of the calf can occur in the pediatric population secondary to trauma, infection, gastrocnemius strain or rupture, or DVT. Distinguishing between popliteal cyst ruptures and deep vein thrombosis can be challenging due to similarity of presentation. The use of imaging is important for distinguishing ruptured Baker’s cysts from DVTs.^13^ The ability to rule out other causes of calf pain such as DVT, ruptured Baker’s cyst, intramuscular hematoma, cellulitis, superficial thrombophlebitis, and tendon or muscle rupture are all crucial to the overall outcome of the patient.^14^ The literature suggests the use of US and MRI can both be useful in the diagnostic evaluation of Baker’s Cysts. Initial imaging modalities usually consist of US and radiographs due to these methods being non-invasive, easily obtainable, and

Relatively inexpensive. The advantages of MRI in the setting of a Baker’s cyst are providing precise location of the cyst and assessing for internal derangement of the knee structure.^15^ This can be advantageous in the setting of surgical planning, especially near dangerous neurovascular structures such as the popliteal artery. A ruptured Baker’s cyst on MRI shows a collection of fluid within the intermuscular plane which aligns with the site of calf pain (Figure 1). Lyme arthritis typically presents with marked joint effusion but relatively mild pain compared to septic arthritis. Patients are often afebrile or minimally febrile and maintain weight-bearing ability. Synovial WBC counts are elevated but usually lower than in acute pyogenic septic arthritis. In contrast, septic arthritis due to pyogenic organisms presents with acute severe pain, inability to bear weight, high fevers, elevated inflammatory markers, and positive synovial cultures. Distinguishing these entities is critical to avoid unnecessary surgical intervention.

This literature review is based on 5 original studies published between 1995-2022 and focused on the presentation of ruptured Baker’s cysts with an underlying diagnosis of Lyme disease. These studies included 8 cyst formations with subsequent rupture in 8 patients that tested positive for Lyme disease. It is important to note that all patients, in the literature review and our case report, were male with no females reported in the literature. We currently do not have a possible explanation for this finding. Another limitation in this study is also lack of data for exposure length prior to symptom onset.

The etiology of Lyme disease as a cause of bakers cyst needs to have a high level of suspicion in endemic areas. If Lyme disease is not considered as a cause, the bakers cyst may be wrongfully diagnosed due to septic etiology, which may lead to unnecessary surgery. Unfortunately, we found that 3 of 8 cases involved unnecessary surgical irrigation and debridement for suspected septic arthritis early in the patients’ respective disease courses; this could have been avoided with appropriate diagnosis and keeping this possible diagnosis in the differential diagnosis list. Lyme disease is a clinical diagnosis in endemic areas, with many patients presenting with early onset of the characteristic “bull’s eye” rash, fever, myalgia. If untreated, early disseminated Lyme disease may involve the nervous system (Lyme neuroborreliosis) or cardiac tissue (Lyme carditis).^16^ Lyme disease associated with ruptured Baker’s cysts took an average of 5.25 days from time of symptom onset to presentation. Symptoms were fairly consistent with that of non-Lyme disease associated with Baker’s cyst and would include posterior knee pain, knee stiffness, and swelling or a mass behind the knee. When laboratory diagnostic confirmation was acquired, antibiotic regimens were altered to consist of either Ceftriaxone, Amoxicillin, Ampicillin, or Doxycycline, resulting in complete resolution in all patient.

In conclusion, Lyme disease (if leading to Baker’s cyst that eventually rupture) can present with an acute red swollen lower calf simulating the symptoms of other condition like deep venous thrombosis or myositis of the gastrocnemius. Despite the rarity of this condition, it should be kept in mind as it can save the patient from unnecessary surgery, which is why our recommendation is to include Lyme titers in high-risk populations upon acute presentation with a red/swollen posterio calf. For an unknown reason we have found that this condition to occur in male patients. Further studies are required to explain this association.
